# Extended Work Hours for Nursing Staff and the Impact on Nursing Home Quality

**DOI:** 10.1093/geroni/igaf122.1495

**Published:** 2025-12-31

**Authors:** Rohit Pradhan, Akbar Ghiasi, Ganisher Davlyatov, Robert Weech-Maldonado

**Affiliations:** Texas State University, San Marcos, Texas, United States; University of the Incarnate Word, San Antonio, Texas, United States; The University of Oklahoma, Oklahoma City, Oklahoma, United States; The University of Alabama at Birmingham, Birmingham, Alabama, United States

## Abstract

Nursing staff—including registered nurses (RNs), licensed practical nurses (LPNs), and certified nursing assistants (CNAs)—are the primary caregivers in nursing homes (NHs), and the quality of care largely depends on their adequacy and expertise. Previous studies, primarily conducted in acute care settings, suggest that extended work hours among nursing staff can lead to more medical errors and lower care quality. This study examined the association between extended work hours among nursing staff and NH quality. It utilized multiple secondary datasets, including the Payroll-Based Journal and Care Compare: Five-Star Quality Rating System (Five-Star QRS) (2020-2022). The study focused on the quality star rating (1-5 scale) from the Five-Star QRS as the dependent variable. The independent variable was extended work hours, measured as the percentage of nursing staff exceeding 50 work hours per week, averaged across the year to calculate the annual facility-level rate. A Multivariable ordinal logistic regression with two-way (facility and year-level) fixed effects was employed with appropriate control variables (42,743). Results indicated that extended work hours were associated with lower odds of achieving a higher star rating. Specifically, for RNs, each additional hour was linked to a 1 percentage point decrease in the odds of being in a higher star rating category. For LPNs and CNAs, the decrease was 2 percentage points per additional hour. All results were statistically significant at p < .0001. Policy and managerial efforts should focus on nursing staff retention and recruitment though workload regulations may be required to ensure sustainable, high-quality NH care.

